# (4-Methyl­phen­yl)[3-(5-nitro-2-fur­yl)-1-phenyl-1*H*-pyrazol-4-yl]methanone

**DOI:** 10.1107/S1600536809047217

**Published:** 2009-11-14

**Authors:** Jia Hao Goh, Hoong-Kun Fun, Nithinchandra, B. Kalluraya

**Affiliations:** a X-ray Crystallography Unit, School of Physics, Universiti Sains Malaysia, 11800 USM, Penang, Malaysia; b Department of Studies in Chemistry, Mangalore University, Mangalagangotri, Mangalore 574 199, India

## Abstract

In the title pyrazole compound, C_21_H_15_N_3_O_4_, an intra­molecular C—H⋯O hydrogen bond generates an *S*(7) ring motif. The essentially planar furan and pyrazole rings [maximum atomic deviations of 0.011 (2) and 0.006 (2) Å, respectively] make a dihedral angle of 9.21 (11)°. The nitro group is approximately coplanar with the attached furan ring, as indicated by the dihedral angle of 4.5 (2)°. In the crystal structure, inter­molecular C—H⋯O inter­actions form bifurcated hydrogen bonds, generating *R* ^1^_2_(7) ring motifs. These hydrogen bonds link the mol­ecules into infinite chains along the *a* axis. The crystal structure is further stabilized by weak inter­molecular π–π inter­actions [centroid–centroid distance = 3.4118 (10) Å].

## Related literature

For general background to and applications of the title compound, see: Hegde *et al.* (2006 ▶); Kalluraya *et al.* (1994 ▶); Rai & Kalluraya (2006 ▶); Rai *et al.* (2008 ▶). For hydrogen-bond motifs, see: Bernstein *et al.* (1995 ▶). For bond-length data, see: Allen *et al.* (1987 ▶). For the stability of the temperature controller used for the data collection, see: Cosier & Glazer (1986 ▶).
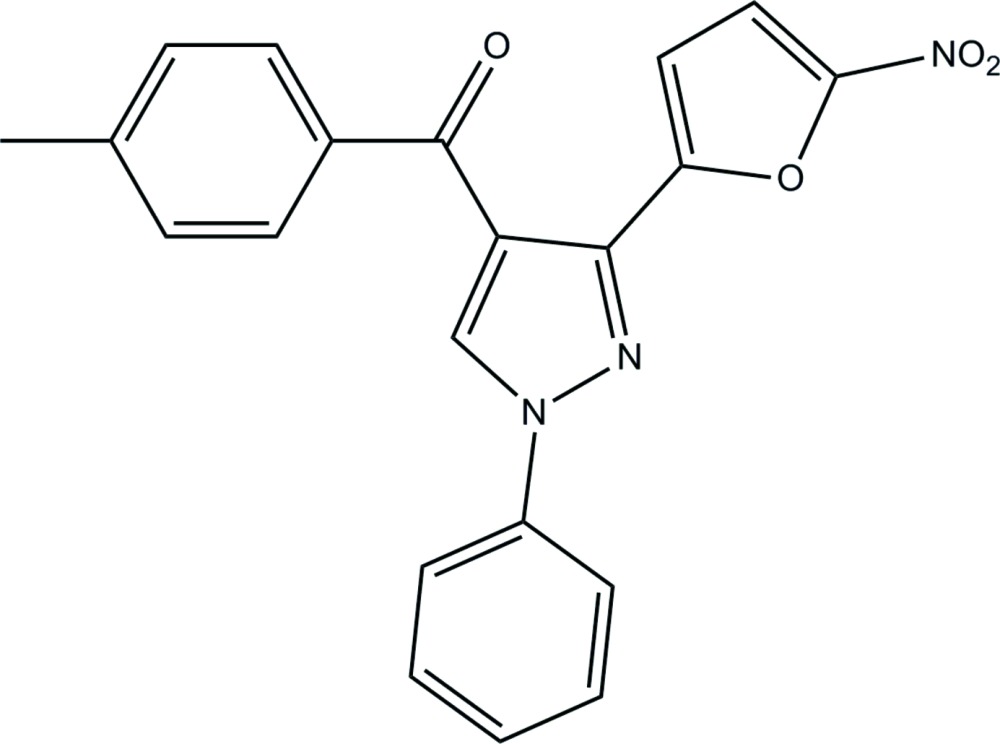


## Experimental

### 

#### Crystal data

C_21_H_15_N_3_O_4_
*M* *_r_* = 373.36
Monoclinic, 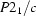

*a* = 11.3859 (2) Å
*b* = 7.5746 (2) Å
*c* = 21.0008 (4) Å
β = 107.202 (1)°
*V* = 1730.17 (6) Å^3^
*Z* = 4
Mo *K*α radiation
μ = 0.10 mm^−1^
*T* = 100 K
0.19 × 0.18 × 0.10 mm

#### Data collection

Bruker SMART APEXII CCD area-detector diffractometer
Absorption correction: multi-scan (**SADABS**; Bruker, 2005 ▶) *T*_min_ = 0.981, *T*_max_ = 0.990
22336 measured reflections
5085 independent reflections
2678 reflections with *I* > 2σ(*I*)
*R*_int_ = 0.085

#### Refinement

*R*[*F* ^2^ > 2σ(*F* ^2^)] = 0.067
*wR*(*F* ^2^) = 0.135
*S* = 1.02
5085 reflections
254 parameters
H-atom parameters constrained
Δρ_max_ = 0.27 e Å^−3^
Δρ_min_ = −0.28 e Å^−3^

### 

Data collection: *APEX2* (Bruker, 2005 ▶); cell refinement: *SAINT* (Bruker, 2005 ▶); data reduction: *SAINT*; program(s) used to solve structure: *SHELXTL* (Sheldrick, 2008 ▶); program(s) used to refine structure: *SHELXTL*; molecular graphics: *SHELXTL*; software used to prepare material for publication: *SHELXTL* and *PLATON* (Spek, 2009 ▶).

## Supplementary Material

Crystal structure: contains datablocks global, I. DOI: 10.1107/S1600536809047217/fj2257sup1.cif

Structure factors: contains datablocks I. DOI: 10.1107/S1600536809047217/fj2257Isup2.hkl

Additional supplementary materials:  crystallographic information; 3D view; checkCIF report

## Figures and Tables

**Table 1:** Hydrogen-bond geometry (Å, °)

*D*—H⋯*A*	*D*—H	H⋯*A*	*D*⋯*A*	*D*—H⋯*A*
C11—H11*A*⋯O2	0.93	2.24	2.902 (2)	128
C14—H14*A*⋯O3^i^	0.93	2.55	3.467 (2)	168
C20—H20*A*⋯O3^i^	0.93	2.46	3.373 (3)	166

Symmetry code: (i) 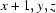.

**Fig. 1.**
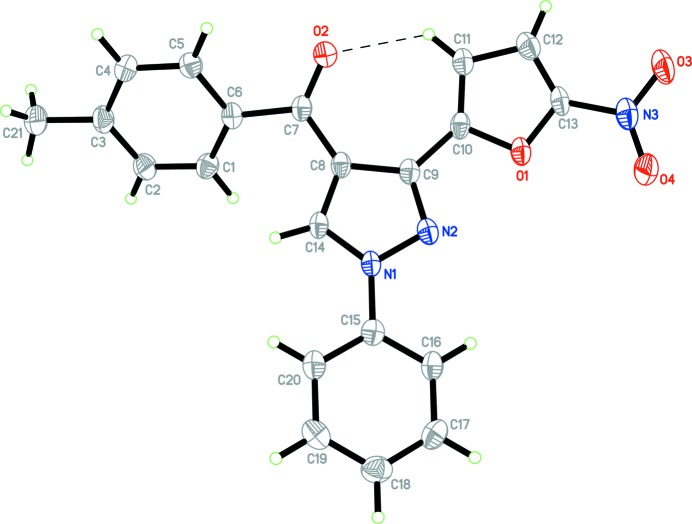
The molecular structure of the title compound, showing 50% probability displacement ellipsoids for non-H atoms and the atom-numbering scheme. An intramolecular hydrogen bond is shown as dashed line.

**Fig. 2.**
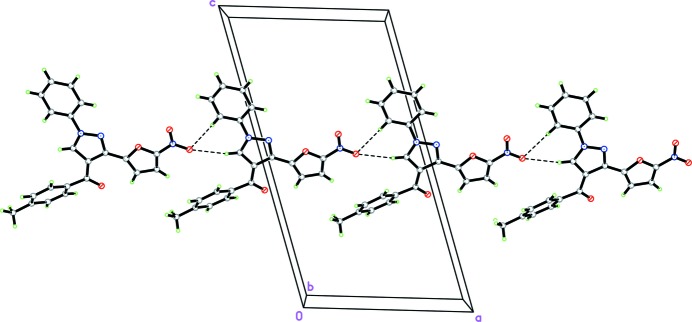
The crystal structure of the title compound, viewed along the b axis, showing one dimensional infinite chains along the a axis. Intermolecular hydrogen bonds are shown as dashed lines.

## supplementary crystallographic information

### Comment

The pyrazole nucleus constitutes an interesting class of organic compound with diverse chemical applications. They possess anti-pyretic, anti-tumor, tranquilizing and herbicidal activities. Sydnones are easily accessible aromatic compounds and versatile synthetic intermediates with a masked azomethine imine unit. The 1,3-dipolar cycloaddition reaction with various dipolarophiles offers a convenient synthetic route for the preparation of pyrazole derivatives and has been studied extensively (Rai & Kalluraya, 2006; Rai *et al.*, 2008).

The incorporation of 5-nitrofuran moiety into various heterocyclic systems has found to increase their biological activities. We have reported a few heterocyclic systems carrying 5-nitrofuran moiety as potent anti-microbial agents (Hegde *et al.*, 2006). In continuation of our studies on 1,3-dipolar cycloaddition reactions of sydnones with dipolarophiles carrying nitrofuran moiety (Kalluraya *et al.*, 1994), we herein report the synthesis of this new pyrazole.

In the title pyrazole compound, an intramolecular C11—H11A···O2 hydrogen bond (Table 1) generates a seven-membered ring, producing an *S*(7) ring motif (Fig. 1, Bernstein *et al.*, 1995). The furan (C10-C13/O1) and pyrazole (C8/C9/N2/N1/C14) rings are essentially planar, with maximum deviations of 0.011 (2) and 0.006 (2) Å, respectively, for atoms C10 and N2. These two rings are slightly twisted to one another, making a dihedral angle of 9.21 (11)° between them. The nitro group is approximately coplanar with the attached furan ring, as shown by the dihedral angle formed between the mean plane through N3/O3/O4 and the C10-C13/O1 furan ring of 4.5 (2)°. The bond lengths (Allen *et al.*, 1987) and angles observed are within normal ranges.

In the crystal structure (Fig. 2), intermolecular C14—H14A···O3 and C20—H20A···O3 interactions (Table 1) form bifurcated acceptor hydrogen bonds which generate *R*^1^_2_(7) ring motifs. These hydrogen bonds link the molecules into one-dimensional infinite chains along the *a* axis. The crystal structure is further stabillized by weak intermolecular π–π interactions [*Cg*1···*Cg*1 = 3.4118 (10) Å; *Cg*1 is the centroid of the C8/C9/N2/N1/C14 pyrazole ring].

### Experimental

3-Phenyl sydnone (0.01 mol) and 1-(*p*-methylphenyl)-3-(5-nitro-2-furyl)-2-propyn-1-one (0.01 mol) were dissolved in 10 ml dry xylene and refluxed for 4 h. After completion of the reaction, the solvent was removed by distillation under reduced pressure. The crude product obtained was purified by recrystallization from ethanol and DMF mixture. The solid obtained was collected by filtration, washed with ethanol and dried. Single crystals suitable for X-ray analysis were obtained from a 1:2 mixture of DMF and ethanol by slow evaporation.

### Refinement

All the hydrogen atoms were placed in their calculated positions, with C—H = 0.93 – 0.96 Å, and refined using a riding model, with *U*_iso_ = 1.2 or 1.5 *U*_eq_(C). A rotating group model was used for the methyl group.

### Crystal data

**Table:**

C_21_H_15_N_3_O_4_	*F*(000) = 776
*M**_r_* = 373.36	*D*_x_ = 1.433 Mg m^−^^3^
Monoclinic, *P*2_1_/*c*	Mo *K*α radiation, λ = 0.71073 Å
Hall symbol: -P 2ybc	Cell parameters from 2474 reflections
*a* = 11.3859 (2) Å	θ = 3.3–30.1°
*b* = 7.5746 (2) Å	µ = 0.10 mm^−^^1^
*c* = 21.0008 (4) Å	*T* = 100 K
β = 107.202 (1)°	Block, brown
*V* = 1730.17 (6) Å^3^	0.19 × 0.18 × 0.10 mm
*Z* = 4	

### Data collection

**Table:**

Bruker SMART APEXII CCD area-detector diffractometer	5085 independent reflections
Radiation source: fine-focus sealed tube	2678 reflections with *I* > 2σ(*I*)
graphite	*R*_int_ = 0.085
φ and ω scans	θ_max_ = 30.1°, θ_min_ = 2.0°
Absorption correction: multi-scan (*SADABS*; Bruker, 2005)	*h* = −15→16
*T*_min_ = 0.981, *T*_max_ = 0.990	*k* = −10→9
22336 measured reflections	*l* = −29→29

### Refinement

**Table:**

Refinement on *F*^2^	Primary atom site location: structure-invariant direct methods
Least-squares matrix: full	Secondary atom site location: difference Fourier map
*R*[*F*^2^ > 2σ(*F*^2^)] = 0.067	Hydrogen site location: inferred from neighbouring sites
*wR*(*F*^2^) = 0.135	H-atom parameters constrained
*S* = 1.02	*w* = 1/[σ^2^(*F*_o_^2^) + (0.0519*P*)^2^ + 0.0761*P*] where *P* = (*F*_o_^2^ + 2*F*_c_^2^)/3
5085 reflections	(Δ/σ)_max_ < 0.001
254 parameters	Δρ_max_ = 0.27 e Å^−^^3^
0 restraints	Δρ_min_ = −0.28 e Å^−^^3^

### Special details

**Table:**

Experimental. The crystal was placed in the cold stream of an Oxford Cyrosystems Cobra open-flow nitrogen cryostat (Cosier & Glazer, 1986) operating at 100.0 (1)K.
Geometry. All esds (except the esd in the dihedral angle between two l.s. planes) are estimated using the full covariance matrix. The cell esds are taken into account individually in the estimation of esds in distances, angles and torsion angles; correlations between esds in cell parameters are only used when they are defined by crystal symmetry. An approximate (isotropic) treatment of cell esds is used for estimating esds involving l.s. planes.
Refinement. Refinement of F^2^ against ALL reflections. The weighted R-factor wR and goodness of fit S are based on F^2^, conventional R-factors R are based on F, with F set to zero for negative F^2^. The threshold expression of F^2^ > 2sigma(F^2^) is used only for calculating R-factors(gt) etc. and is not relevant to the choice of reflections for refinement. R-factors based on F^2^ are statistically about twice as large as those based on F, and R- factors based on ALL data will be even larger.

### Fractional atomic coordinates and isotropic or equivalent isotropic displacement parameters (Å^2^)

**Table:**

	*x*	*y*	*z*	*U*_iso_*/*U*_eq_	
O1	−0.26961 (11)	0.68826 (17)	−0.02101 (6)	0.0275 (3)	
O2	0.05600 (11)	0.98467 (18)	0.11675 (6)	0.0286 (3)	
O3	−0.56846 (12)	0.7483 (2)	−0.01746 (8)	0.0464 (4)	
O4	−0.49541 (13)	0.5852 (2)	−0.08230 (8)	0.0473 (4)	
N1	0.04825 (13)	0.6498 (2)	−0.06270 (7)	0.0219 (4)	
N2	−0.06690 (14)	0.6518 (2)	−0.05573 (8)	0.0245 (4)	
N3	−0.48241 (15)	0.6877 (2)	−0.03543 (9)	0.0340 (4)	
C1	0.33869 (18)	0.7702 (3)	0.12408 (9)	0.0284 (5)	
H1A	0.3085	0.6624	0.1049	0.034*	
C2	0.46370 (18)	0.7905 (3)	0.15579 (10)	0.0320 (5)	
H2A	0.5160	0.6946	0.1585	0.038*	
C3	0.51237 (18)	0.9510 (3)	0.18361 (10)	0.0310 (5)	
C4	0.43195 (18)	1.0917 (3)	0.17911 (9)	0.0294 (5)	
H4A	0.4628	1.2009	0.1965	0.035*	
C5	0.30696 (18)	1.0725 (3)	0.14930 (9)	0.0269 (5)	
H5A	0.2546	1.1675	0.1480	0.032*	
C6	0.25869 (17)	0.9108 (3)	0.12104 (9)	0.0248 (4)	
C7	0.12233 (17)	0.8988 (2)	0.09159 (9)	0.0222 (4)	
C8	0.06949 (16)	0.7914 (2)	0.03174 (9)	0.0222 (4)	
C9	−0.05508 (16)	0.7393 (2)	0.00105 (9)	0.0220 (4)	
C10	−0.16698 (17)	0.7701 (2)	0.01993 (9)	0.0236 (4)	
C11	−0.19765 (17)	0.8688 (3)	0.06673 (10)	0.0281 (5)	
H11A	−0.1443	0.9338	0.1007	0.034*	
C12	−0.32650 (18)	0.8533 (3)	0.05368 (10)	0.0327 (5)	
H12A	−0.3752	0.9068	0.0767	0.039*	
C13	−0.36327 (17)	0.7451 (3)	0.00098 (10)	0.0274 (5)	
C14	0.13067 (17)	0.7302 (2)	−0.01160 (9)	0.0220 (4)	
H14A	0.2143	0.7425	−0.0064	0.026*	
C15	0.06792 (17)	0.5688 (2)	−0.12035 (9)	0.0232 (4)	
C16	−0.02628 (18)	0.4746 (3)	−0.16354 (9)	0.0273 (5)	
H16A	−0.1025	0.4664	−0.1559	0.033*	
C17	−0.00643 (19)	0.3924 (3)	−0.21841 (10)	0.0331 (5)	
H17A	−0.0693	0.3275	−0.2473	0.040*	
C18	0.10610 (19)	0.4062 (3)	−0.23042 (10)	0.0345 (5)	
H18A	0.1190	0.3516	−0.2674	0.041*	
C19	0.19937 (19)	0.5017 (3)	−0.18716 (10)	0.0328 (5)	
H19A	0.2751	0.5111	−0.1953	0.039*	
C20	0.18140 (18)	0.5839 (3)	−0.13170 (10)	0.0278 (5)	
H20A	0.2445	0.6481	−0.1026	0.033*	
C21	0.64761 (18)	0.9736 (3)	0.21836 (11)	0.0425 (6)	
H21A	0.6768	1.0784	0.2022	0.064*	
H21B	0.6916	0.8729	0.2095	0.064*	
H21C	0.6607	0.9838	0.2655	0.064*	

### Atomic displacement parameters (Å^2^)

**Table:**

	*U*^11^	*U*^22^	*U*^33^	*U*^12^	*U*^13^	*U*^23^
O1	0.0179 (7)	0.0278 (8)	0.0350 (8)	−0.0026 (6)	0.0053 (6)	0.0027 (6)
O2	0.0245 (8)	0.0303 (8)	0.0307 (7)	−0.0007 (6)	0.0078 (6)	−0.0007 (6)
O3	0.0191 (8)	0.0648 (12)	0.0557 (10)	−0.0010 (8)	0.0118 (8)	−0.0040 (9)
O4	0.0270 (9)	0.0471 (10)	0.0633 (11)	−0.0079 (8)	0.0062 (8)	−0.0162 (9)
N1	0.0163 (8)	0.0217 (9)	0.0264 (8)	−0.0011 (7)	0.0045 (7)	0.0028 (7)
N2	0.0185 (9)	0.0234 (9)	0.0315 (9)	0.0000 (7)	0.0073 (7)	0.0040 (7)
N3	0.0214 (10)	0.0361 (11)	0.0429 (11)	−0.0028 (8)	0.0069 (9)	0.0053 (9)
C1	0.0259 (11)	0.0278 (12)	0.0283 (11)	−0.0008 (10)	0.0031 (9)	0.0010 (9)
C2	0.0228 (11)	0.0345 (13)	0.0354 (12)	0.0051 (10)	0.0035 (10)	0.0009 (10)
C3	0.0213 (11)	0.0412 (14)	0.0279 (11)	−0.0021 (10)	0.0036 (9)	−0.0005 (10)
C4	0.0262 (11)	0.0302 (12)	0.0292 (11)	−0.0049 (10)	0.0040 (9)	−0.0017 (9)
C5	0.0244 (11)	0.0279 (12)	0.0279 (10)	0.0009 (9)	0.0070 (9)	0.0018 (9)
C6	0.0205 (10)	0.0289 (11)	0.0242 (10)	−0.0020 (9)	0.0052 (9)	0.0042 (9)
C7	0.0202 (10)	0.0216 (11)	0.0237 (10)	−0.0023 (8)	0.0046 (8)	0.0055 (8)
C8	0.0180 (10)	0.0207 (10)	0.0259 (10)	−0.0008 (8)	0.0033 (8)	0.0051 (8)
C9	0.0192 (10)	0.0184 (10)	0.0260 (10)	0.0010 (8)	0.0031 (8)	0.0058 (8)
C10	0.0187 (10)	0.0206 (11)	0.0283 (10)	−0.0031 (8)	0.0021 (9)	0.0059 (8)
C11	0.0205 (11)	0.0307 (12)	0.0321 (11)	−0.0018 (9)	0.0062 (9)	0.0013 (9)
C12	0.0242 (11)	0.0372 (13)	0.0386 (12)	−0.0002 (10)	0.0124 (10)	0.0007 (10)
C13	0.0149 (10)	0.0299 (12)	0.0363 (11)	−0.0010 (9)	0.0059 (9)	0.0066 (10)
C14	0.0164 (10)	0.0212 (11)	0.0251 (10)	−0.0016 (8)	0.0013 (8)	0.0052 (8)
C15	0.0229 (10)	0.0205 (10)	0.0240 (10)	0.0020 (9)	0.0034 (9)	0.0060 (8)
C16	0.0209 (11)	0.0295 (12)	0.0281 (10)	−0.0006 (9)	0.0020 (9)	0.0042 (9)
C17	0.0316 (13)	0.0349 (13)	0.0267 (11)	−0.0021 (10)	−0.0006 (10)	0.0000 (10)
C18	0.0383 (13)	0.0374 (13)	0.0269 (11)	0.0072 (11)	0.0083 (10)	0.0007 (10)
C19	0.0296 (12)	0.0347 (13)	0.0365 (12)	0.0051 (10)	0.0136 (10)	0.0067 (10)
C20	0.0217 (11)	0.0275 (11)	0.0319 (11)	−0.0009 (9)	0.0042 (9)	0.0030 (9)
C21	0.0238 (12)	0.0503 (16)	0.0482 (14)	−0.0021 (11)	0.0024 (11)	−0.0084 (12)

### Geometric parameters (Å, °)

**Table:**

O1—C13	1.352 (2)	C8—C14	1.380 (2)
O1—C10	1.377 (2)	C8—C9	1.429 (2)
O2—C7	1.229 (2)	C9—C10	1.461 (2)
O3—N3	1.2377 (19)	C10—C11	1.360 (3)
O4—N3	1.228 (2)	C11—C12	1.415 (3)
N1—C14	1.345 (2)	C11—H11A	0.9300
N1—N2	1.3617 (19)	C12—C13	1.341 (3)
N1—C15	1.433 (2)	C12—H12A	0.9300
N2—C9	1.336 (2)	C14—H14A	0.9300
N3—C13	1.414 (2)	C15—C16	1.381 (3)
C1—C2	1.390 (3)	C15—C20	1.386 (2)
C1—C6	1.391 (3)	C16—C17	1.386 (3)
C1—H1A	0.9300	C16—H16A	0.9300
C2—C3	1.391 (3)	C17—C18	1.381 (3)
C2—H2A	0.9300	C17—H17A	0.9300
C3—C4	1.390 (3)	C18—C19	1.380 (3)
C3—C21	1.506 (3)	C18—H18A	0.9300
C4—C5	1.383 (3)	C19—C20	1.388 (3)
C4—H4A	0.9300	C19—H19A	0.9300
C5—C6	1.400 (3)	C20—H20A	0.9300
C5—H5A	0.9300	C21—H21A	0.9600
C6—C7	1.494 (3)	C21—H21B	0.9600
C7—C8	1.468 (3)	C21—H21C	0.9600
			
C13—O1—C10	104.64 (15)	O1—C10—C9	113.95 (16)
C14—N1—N2	112.07 (14)	C10—C11—C12	106.71 (18)
C14—N1—C15	128.45 (15)	C10—C11—H11A	126.6
N2—N1—C15	119.47 (15)	C12—C11—H11A	126.6
C9—N2—N1	104.81 (15)	C13—C12—C11	105.26 (17)
O4—N3—O3	124.09 (18)	C13—C12—H12A	127.4
O4—N3—C13	119.78 (17)	C11—C12—H12A	127.4
O3—N3—C13	116.12 (18)	C12—C13—O1	113.05 (17)
C2—C1—C6	120.08 (19)	C12—C13—N3	130.51 (18)
C2—C1—H1A	120.0	O1—C13—N3	116.41 (18)
C6—C1—H1A	120.0	N1—C14—C8	108.03 (16)
C1—C2—C3	121.47 (19)	N1—C14—H14A	126.0
C1—C2—H2A	119.3	C8—C14—H14A	126.0
C3—C2—H2A	119.3	C16—C15—C20	120.81 (18)
C4—C3—C2	117.98 (19)	C16—C15—N1	119.41 (16)
C4—C3—C21	120.30 (19)	C20—C15—N1	119.78 (17)
C2—C3—C21	121.72 (19)	C15—C16—C17	119.49 (17)
C5—C4—C3	121.27 (19)	C15—C16—H16A	120.3
C5—C4—H4A	119.4	C17—C16—H16A	120.3
C3—C4—H4A	119.4	C18—C17—C16	120.4 (2)
C4—C5—C6	120.43 (18)	C18—C17—H17A	119.8
C4—C5—H5A	119.8	C16—C17—H17A	119.8
C6—C5—H5A	119.8	C19—C18—C17	119.62 (19)
C1—C6—C5	118.74 (18)	C19—C18—H18A	120.2
C1—C6—C7	123.94 (18)	C17—C18—H18A	120.2
C5—C6—C7	117.27 (17)	C18—C19—C20	120.77 (18)
O2—C7—C8	120.88 (17)	C18—C19—H19A	119.6
O2—C7—C6	119.03 (17)	C20—C19—H19A	119.6
C8—C7—C6	120.04 (16)	C15—C20—C19	118.91 (19)
C14—C8—C9	103.72 (16)	C15—C20—H20A	120.5
C14—C8—C7	126.29 (17)	C19—C20—H20A	120.5
C9—C8—C7	129.78 (16)	C3—C21—H21A	109.5
N2—C9—C8	111.36 (15)	C3—C21—H21B	109.5
N2—C9—C10	117.12 (17)	H21A—C21—H21B	109.5
C8—C9—C10	131.49 (18)	C3—C21—H21C	109.5
C11—C10—O1	110.30 (15)	H21A—C21—H21C	109.5
C11—C10—C9	135.64 (18)	H21B—C21—H21C	109.5
			
C14—N1—N2—C9	1.1 (2)	N2—C9—C10—O1	6.0 (2)
C15—N1—N2—C9	−178.35 (15)	C8—C9—C10—O1	−176.27 (17)
C6—C1—C2—C3	−1.6 (3)	O1—C10—C11—C12	−1.9 (2)
C1—C2—C3—C4	0.3 (3)	C9—C10—C11—C12	174.0 (2)
C1—C2—C3—C21	179.66 (18)	C10—C11—C12—C13	0.9 (2)
C2—C3—C4—C5	1.4 (3)	C11—C12—C13—O1	0.4 (2)
C21—C3—C4—C5	−177.92 (17)	C11—C12—C13—N3	−177.31 (19)
C3—C4—C5—C6	−1.9 (3)	C10—O1—C13—C12	−1.5 (2)
C2—C1—C6—C5	1.2 (3)	C10—O1—C13—N3	176.56 (16)
C2—C1—C6—C7	−176.31 (17)	O4—N3—C13—C12	−179.2 (2)
C4—C5—C6—C1	0.5 (3)	O3—N3—C13—C12	1.7 (3)
C4—C5—C6—C7	178.19 (16)	O4—N3—C13—O1	3.2 (3)
C1—C6—C7—O2	144.79 (18)	O3—N3—C13—O1	−175.94 (16)
C5—C6—C7—O2	−32.7 (2)	N2—N1—C14—C8	−0.6 (2)
C1—C6—C7—C8	−37.8 (3)	C15—N1—C14—C8	178.73 (16)
C5—C6—C7—C8	144.68 (17)	C9—C8—C14—N1	−0.1 (2)
O2—C7—C8—C14	160.61 (18)	C7—C8—C14—N1	−175.08 (17)
C6—C7—C8—C14	−16.7 (3)	C14—N1—C15—C16	172.41 (18)
O2—C7—C8—C9	−13.1 (3)	N2—N1—C15—C16	−8.3 (3)
C6—C7—C8—C9	169.56 (18)	C14—N1—C15—C20	−6.8 (3)
N1—N2—C9—C8	−1.1 (2)	N2—N1—C15—C20	172.55 (16)
N1—N2—C9—C10	177.03 (15)	C20—C15—C16—C17	0.8 (3)
C14—C8—C9—N2	0.8 (2)	N1—C15—C16—C17	−178.34 (17)
C7—C8—C9—N2	175.53 (17)	C15—C16—C17—C18	−0.9 (3)
C14—C8—C9—C10	−177.03 (19)	C16—C17—C18—C19	0.4 (3)
C7—C8—C9—C10	−2.3 (3)	C17—C18—C19—C20	0.0 (3)
C13—O1—C10—C11	2.0 (2)	C16—C15—C20—C19	−0.4 (3)
C13—O1—C10—C9	−174.79 (15)	N1—C15—C20—C19	178.79 (17)
N2—C9—C10—C11	−169.7 (2)	C18—C19—C20—C15	−0.1 (3)
C8—C9—C10—C11	8.0 (4)		

### Hydrogen-bond geometry (Å, °)

**Table:**

*D*—H···*A*	*D*—H	H···*A*	*D*···*A*	*D*—H···*A*
C11—H11A···O2	0.93	2.24	2.902 (2)	128
C14—H14A···O3^i^	0.93	2.55	3.467 (2)	168
C20—H20A···O3^i^	0.93	2.46	3.373 (3)	166

Symmetry codes: (i) *x*+1, *y*, *z*.
